# High-Throughput Biofilm Assay to Investigate Bacterial Interactions with Surface Topographies

**DOI:** 10.1021/acsabm.2c00367

**Published:** 2022-07-11

**Authors:** Sang Won Lee, Erick L. Johnson, J. Alex Chediak, Hainsworth Shin, Yi Wang, K. Scott Phillips, Dacheng Ren

**Affiliations:** † Department of Biomedical and Chemical Engineering, Syracuse University, Syracuse, New York 13244, United States; ‡ Office of Medical Products and Tobacco, Center for Devices and Radiological Health, Office of Science and Engineering Laboratories, Division of Biology, Chemistry, and Materials Science, United States Food and Drug Administration, Silver Spring, Maryland 20993, United States; § Mechanical and Industrial Engineering, Montana State University, Bozeman, Montana 59717, United States; ∥ Department of Mathematical Sciences, California Baptist University, Riverside, California 92504, United States; ⊥ Department of Civil and Environmental Engineering, Syracuse University, Syracuse, New York 13244, United States; # Department of Biology, Syracuse University, Syracuse, New York 13244, United States

**Keywords:** biofilm, high-throughput assay, texture, topography, breast implant, BIA-ALCL

## Abstract

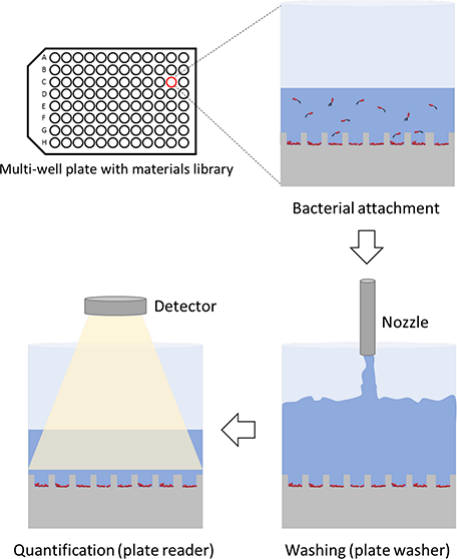

The specific topography of biomaterials plays an important role in their biological interactions with cells and thus the safety of medical implants. Antifouling materials can be engineered with topographic features to repel microbes. Meanwhile, undesired topographies of implants can cause complications such as breast implant-associated anaplastic large cell lymphoma (BIA-ALCL). While the cause of BIA-ALCL is not well understood, it is speculated that textured surfaces are prone to bacterial biofilm formation as a contributing factor. To guide the design of safer biomaterials and implants, quantitative screening approaches are needed to assess bacterial adhesion to different topographic surface features. Here we report the development of a high-throughput microplate biofilm assay for such screening. The assay was used to test a library of polydimethylsiloxane (PDMS) textures composed of varying sizes of recessive features and distances between features including those in the range of breast implant textures. Outliers of patterns prone to bacterial adhesion were further studied using real-time confocal fluorescence microscopy. The results from these analyses revealed that surface area itself is a poor predictor for adhesion, while the size and spacing of topographic features play an important role. This high-throughput biofilm assay can be applied to studying bacteria–material interactions and rational development of materials that inhibit bacterial colonization.

## 1 Introduction

Rational design of safe medical devices requires a good understanding of how surface texture affects bacteria–material interactions. While some medical devices such as surgical instruments are engineered with smooth surfaces to ensure cleanability, others such as breast implants are intentionally textured to increase tissue interactions. The texture of medical device surfaces can also impact bacterial adhesion and colonization. Numerous antifouling topographies have been reported. Meanwhile, poorly suited topographies can potentially increase the risk of infection or associated adverse events, such as inflammation.^1−3^

Textured breast implants provide a recent, high-profile example of adverse events related to surface texture. In 2011, the U.S. Food and Drug Administration (FDA) first communicated a potential association between silicone gel-filled breast implants and incidence of anaplastic large cell lymphoma, termed breast implant-associated anaplastic large cell lymphoma (BIA-ALCL).^4^ The incidence of BIA-ALCL was found to be higher for textured implants compared to smooth surface implants, resulting in regulatory action in 2019.^5^ While the cause of BIA-ALCL is not well understood at this time, it has been hypothesized that bacterial biofilms on the implants may be a contributing factor.^6−9^ In particular, it was hypothesized that larger surface areas may lead to greater microbial bioburden, increasing the probability that chronic antigen stimulation might cross a hypothetical threshold for the onset of ALCL.^9^ Meanwhile, some investigators found no difference in the relative abundance of Gram-negative bacteria between BIA-ALCL and control specimens.^10^ Other potential triggers include breast implant debris,^11,12^ differences in the microbiome among patients,^13^ chemical leachates,^14^ and hydrodynamic forces associated with texture.^15^ To understand the link between surface texture, infection, and the pathobiology of BIA-ALCL as well as to improve future designs, it is important to know how surface texture affects bacterial adhesion.

Many efforts to relate safety risk to the texture of medical devices have focused on surface roughness. It is also important to consider more unique pattern-specific features of surface topography such as size scales, internal/external edges, overhangs, spacing, and depth. A better understanding of pattern-specific bacterial interactions is necessary to discern how quantitative measurements will perform across a variety of different qualitative textures and size scales—and whether they can be generalized to novel, untested features.

This requires testing of a large number of samples and quantitative comparison of biofilm on different topographic patterns. Current methods of studying bacterial adhesion are low throughput and have large variations in results between researchers and different laboratories due to manual handling of samples—especially manual sample wash steps. In response to these limitations, we developed a reproducible and controllable high-throughput method and optimized the parameters for screening of material libraries. We validated this approach using a library of polydimethylsiloxane (PDMS) surfaces composed of varying sizes of recessive topographic features and distances between features. The patterned surfaces are secured to 96-well plates and consistent washing conditions are applied to the wells using a plate washer. The method was modeled with computational simulation, and quantitative results were confirmed with confocal microscopy.

## 2 Materials and Methods

### 2.1 PDMS Surface Fabrication

To obtain polydimethylsiloxane surfaces with topographic patterns of interest, a Si wafer was fabricated at Cornell NanoScale Science & Facility (CNF) using photolithography (Supporting Information Figure S1a). Briefly, the square-shaped pattern features with different side lengths and spacing were designed by L-edit computer-aided design (CAD) software. To investigate the effects of feature size on bacterial adhesion, we varied the side length (*S*) from 2 to 300 μm and the distance (*D*) between features from 2 to 100 μm (Figure S1b). All patterns had a depth of 10 μm. To fabricate the topographic patterns on the Si wafer, a Cr deposited quartz mask including CAD designed topographic patterns was first created. A positive photoresist (PR) was coated on the mask. Then it was exposed by UV using a DWL 2000 Heidelberg mask writer (Heidelberg Instruments Mikrotechnik GmbH, Heidelberg, Germany) based on the CAD file. Only the photoreacted area was exposed to PR developer and Cr etchant. The rest of the PR layer was stripped by *N*-methyl-2-pyrrolidone (NMP) and tetramethylammonium hydroxide (TMAH) based cleaning solution for 30 min in a 60 °C hot bath.

To create features on a silicon (Si) wafer, a 30–50 nm P20 adhesion layer and a 1.8–2.5 μm positive PR layer (S1813) were deposited first using a spin coater at 2,000 rpm for 60 s. An ABM contact aligner (1:1 ratio photolithography; ABM USA Inc., San Jose, CA, USA) was used to draw features on the Si wafer by exposing to UV light through the Cr mask followed by a development process using the TMAH-based cleaning solution. The developed Si wafer was then etched to 10 μm depth by a deep reactive ion Si etcher (DRIE; Plasma-Therm LLC, St. Petersburg, FL, USA). A YES Asher (Yield Engineering Systems Inc., Livermore, CA, USA) stripper was used to strip the remaining PR from the etched Si wafer. To ease the peeling of the PDMS layer from the Si wafer, a surface of the etched Si wafer was made hydrophobic by molecular vapor deposition (MVD; Applied Microstructures, San Jose, CA, USA) of fluoro-octyltrichlorosilane (FOTS).

The patterned Si wafer was then used as a master to fabricate PDMS with designed features.^16^ A mixture of 10:1 weight ratio of Dow Sylgard 184 base and curing agent (The Dow Chemical Co., Midland, MI, USA) was mixed and vacuumed for 1 h to remove air bubbles produced during the chemical reaction of base and curing agent. The vacuumed mixture was then poured on the Si master, spin-coated for 1 min at 50 rpm, and vacuumed again for 1 h to remove all trapped air bubbles inside the features. After 1 h of vacuum, the sample was cured at 60 °C for 2 h and cooled at room temperature for 1 h.

### 2.2 Surface Analysis

PDMS surfaces were also analyzed using scanning electron microscopy (SEM, JEOL Ltd., Tokyo, Japan). The PDMS samples were coated with 10 nm gold (Au) using a sputter coater (Denton Vacuum LLC, Moorestown, NJ, USA).

### 2.3 Rinse Process Stringency Evaluated by Computational Fluid Dynamics

The computational fluid dynamics (CFD) software Siemens’ SimCenter Star-CCM+ (v15.04.010) was used to simulate the rinse process. Steady, two-dimensional, single-phase simulations approximated the rinsing process over two different surface features (S10 D5 and S300 D100) and flat PDMS and were analyzed to visualize vector direction of the flow and shear stress on the surface. A maximum, tangential flow velocity of 1.5 m/s was calculated on the basis of the flow rate (200 μL dispensed at a rate of 800 μL/s), the rinse manifold dimensions, and location (0.7 mm diameter located 13.49 mm above the sample) impacting a partially filled well. This velocity was verified against an unsteady, three-dimensional model of the flat PDMS sample, and the dispenser of the wash plate was off-center of each sample.

### 2.4 Bacterial Strains and Medium

*Escherichia coli* (*E. coli*) RP437/pRSH103 was used as a model strain in this study because *E. coli* strains are common bacteria found in biofilm infections and they can sense and interact with surface topographies by using flagella. *E. coli* has also been found in explant materials from BIA-ALCL patients and other breast implant-associated complications.^7,10,17−19^ As a Gram-negative organism, *E. coli* has lipopolysaccharides (LPSs) in its outer membrane that are a type of endotoxin eliciting inflammatory cytokines. It is not known whether chronic stimulation with LPS leads to BIA-ALCL or other conditions, although this has been proposed as a mechanism by some investigators.^20^ RP437/pRSH103^21^ was grown in tryptic soy broth (TSB; Thermo Fisher Scientific, Waltham, MA, USA) or lysogeny broth (LB)^22^ supplemented with 30 μg/mL of tetracycline (Sigma-Aldrich, St. Louis, MO, USA).

### 2.5 High-Throughput Assay

To quantify the biomass on PDMS surfaces in a high-throughput manner, each PDMS sample was punched with a 6 mm biopsy puncher (Integra Lifesciences, Plainsboro Township, NJ, USA) and transferred into a 96 well plate (Santa Cruz Biotechnology, Inc., Dallas, TX, USA). Each PDMS sample was attached to the bottom of a well using three additional droplets of PDMS mixture, which cover the rest of the well surface and make the PDMS sample stick to the well. After curing at 60 °C for 2 h, the loaded PDMS surfaces were sterilized by UV for 1 h prior to inoculation.

*E. coli* RP437/pRSH103 was used to inoculate biofilm cultures in each well with 100 μL growth medium covering the PDMS sample. The culture was inoculated with a starting optical density (OD) at 600 nm (OD_600_) of 0.1. To remove trapped air bubbles from the PDMS surface, 100 μL sterile phosphate-buffered saline (PBS) was added in each well and vacuumed for 30 min prior to inoculation. The cultures were incubated for 4 h at 37 °C with shaking at 200 rpm for agitation condition and without shaking for static condition.

After incubation, the samples were washed three times with PBS using a plate washer (50TS microplate washer, BioTek, Winooski, VT, USA). There were three washing steps: dispense, hold, and aspirate. The flow rate of dispense and aspiration is adjustable from 200 to 1000 μL/s, and manifold height can move from the top to the bottom of each well. To quantify biomass, the signal of red fluorescent protein (RFP) (excitation, 558 nm; emission, 583 nm) was measured using a plate reader (TECAN infinite M1000, Tecan, Mannedorf, Switzerland). The collection focus was on the center of the well, and the sides do not contribute significantly to the overall signal collected.

### 2.6 Microscopy

To visualize the biomass in 3D, biofilms were analyzed using confocal microscopy (Leica SP8, Leica Camera AG, Wetzlar, Germany) and fluorescence microscopy (Axio Imager M1, Carl Zeiss Inc., Berlin, Germany). To quantify the biomass, Z-stack images with 3D information were obtained by confocal microscopy (upright pattern) and fluorescence microscopy (upside down pattern) followed by quantification using the software COMSTAT.^23^ The experiments were conducted with three biological replicates with five random images taken from each sample.

### 2.7 Correlation between Biomass and Surface Properties

The side length (*S*) of 10 μm deep recessive patterns was varied as 2, 5, 10, 50, 100, 200, and 300 μm. The distance (*D*) between adjacent patterns was varied as 2, 5, 10, 50, and 100 μm. To understand the effect of underlying properties on bacterial adhesion in the PDMS library, the adhesion was plotted against surface parameters such as surface roughness, surface area ratio, and intersection length (sum of bottom side lengths of recessive square patterns).

### 2.8 Statistics

SAS 9.1.3, Windows version (SAS, Cary, NC, USA) was used for all statistical analyses. Results with *p* < 0.05 were considered statistically significant.

## 3 Results

### 3.1 Design of the Topographic Feature Library

To develop and validate the high-throughput assay, we created a library of recessive topographic features relevant to those found on textured breast implants. One of the most common manufacturing methods for textured breast implants is the “salt-loss” method,^9^ which creates a recessive cube-like topography ranging from 10 to 300 μm with surface roughness value between 21.39 and 79.51 μm.^24^ To systematically characterize how variations in such topography affect bacterial attachment, the side length of the 10 μm deep recessive square-shaped patterns was varied to be 2, 5, 10, 50, 100, 200, and 300 μm; and the distance between recessive patterns was varied to be 2, 5, 10, 50, and 100 μm (Table S1). The lower end of this range was chosen to understand differences near the size scale of bacterial cells, while the upper end was chosen to encompass the largest features found on commercial breast implants (Table S1).^24^

### 3.2 Development of a High-Throughput Adhesion Assay

The assays of biofilm formation on topographic features to date are largely low throughput with each sample examined by microscopy.^25−29^ To analyze bacterial adhesion to a large number of samples with different surface features, a high-throughput method was developed in this study. PDMS coupons with topographic features on one side were mounted in 96 well plates, inoculated with fluorescent bacteria, and rinsed after 4 h of incubation, followed by fluorescence quantification using a 96 well plate reader. This method not only improves throughput but also reduces high variability during manual washing of samples often found in measurements of bacterial adhesion and biofilm formation on materials.

To optimize the plate washing process, we first varied the dispense flow rate and manifold height to apply stringency to loosely associated planktonic cells but not remove firmly attached cells. The amount of detected biomass varied in terms of the dispense flow rate (200–800 μL/s) during the washing process (Figure S2). The highest signal intensity with a narrow standard deviation range was observed at the dispense flow rate of 800 μL/s. Other parameters for the plate washer such as the optimal manifold position height during aspiration and dispense process were determined through a similar process.

**Figure 1:**
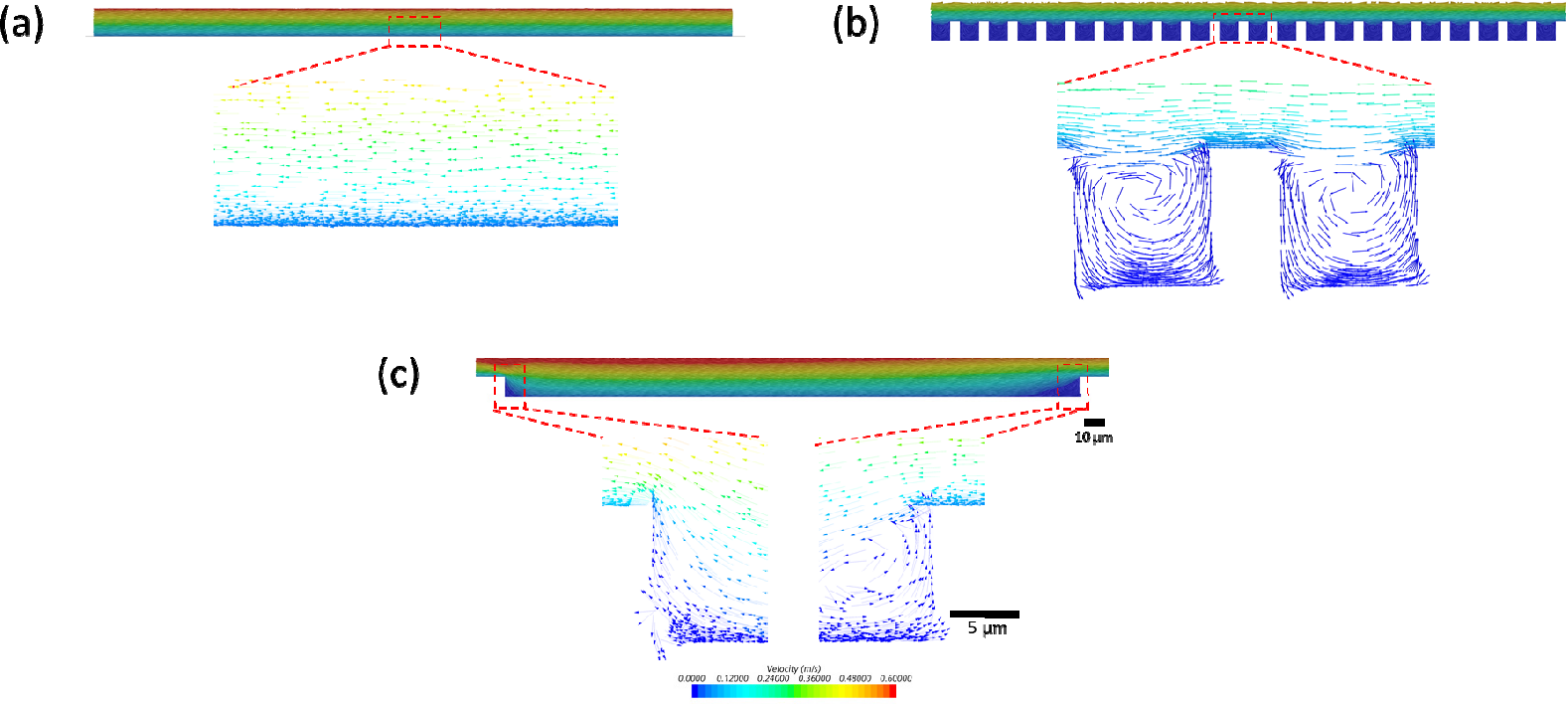
Simulated vector images of rinsing process (flow velocity: 1.5 m/s) of (a) flat PDMS, (b) S10 D5 recessive wells, and (c) S300 D100 recessive wells (scale bar: 10 μm for zoomed-out images and 5 μm for zoomed-in images).

To better understand the shear forces generated by this method, the flow of the optimized rinse profile was then simulated using Star-CCM+ (Figure 1) and shear forces inside and outside of topographic features were compared. Due to computational limitations because of the smallest feature sizes, steady, 2D simulations were used to approximate the flow regimes and largest shear forces that would be observed (Figure 1). The actual parameters of plate washer were used for computational simulation which showed the Reynolds number across the surface is less than 500 (stable laminar). There is no concern about turbulence that would dislodge truly attached biofilm cells in recessive patterns. The maximum wall shear forces inside features were measured for three patterns: flat (0.18 nN), S10 D5 (0.01 nN), and S300 D100 (0.12 nN). These shear forces were calculated from the average, simulated shear stress applied over the surface of a typical *E. coli* cell (area of 3.7 μm^2^).^30−32^ These forces are all below the typical adhesion strength of *E. coli.*, which varies from 0.5 to 24 nN depending on growth stage and material.^33−35^ Thus, the rinse is not expected to dislodge truly attached *E. coli* cells in the patterns. However, the flows generated in all three patterns were adequate to remove loosely bound or planktonic cells. If the shear force was in excess of the adhesion strength, scouring would occur in the center of the sample surfaces. This was not observed. At the other extreme, if the flushing dynamics of the rinse were insufficient to remove excess cells, we would expect to see moving planktonic or loosely attached cells above the substrate. This was also not the case because we observed a single layer of cells attached on the substrate. In summary, the use of a plate washer provided not only greater throughput than manual rinsing but also three key advantages of uniformity, reproducibility, and control (shear force at a level that thoroughly removed loosely bound cells but not the ones firmly attached).

After rinsing, the bacteria that remained on coupons were quantified using a fluorescence plate reader with adjustable focal height (Figure S2). The focus height was varied from 0 to 8000 μm (*Z*-direction). The detector showed the highest signal intensity when positioned at 4,000 μm (Figure S2). The optimized conditions for the plate washer (dispense flow rate 800 μL/s) and the detector position height (4,000 μm) were chosen for further studies using this protocol.

### 3.3 High-Throughput Adhesion Assay Results

**Figure 2:**
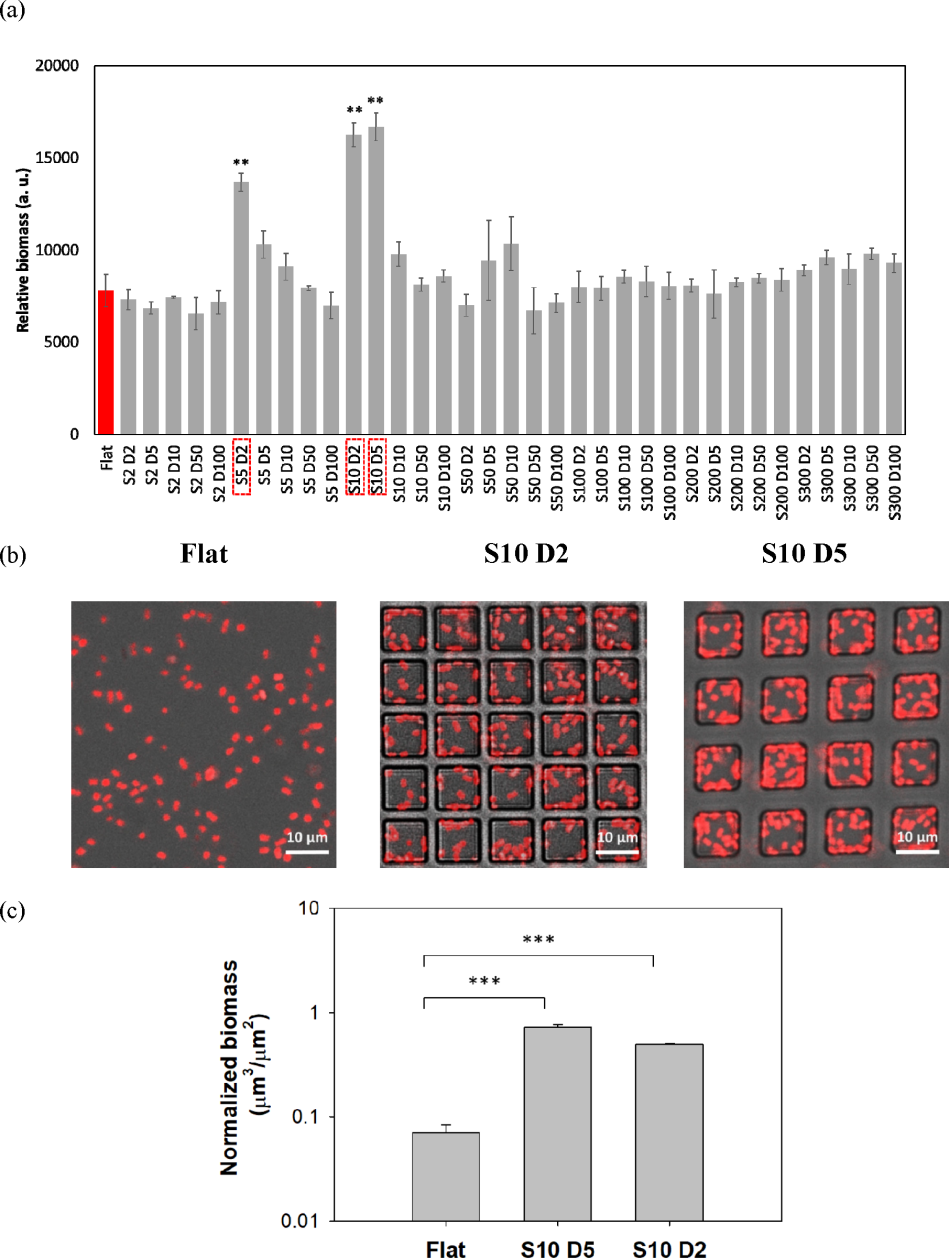
(a) Relative biomass of *E. coli* RP437/pRSH103 on upright patterned PDMS surfaces after 4 h attachment under static condition. Outliers are highlighted. Red bar: flat control. **, *p* < 0.01. (b) Representative fluorescent confocal microscopic images of upright flat, S10 D2, and S10 D5 patterns. Scale bar = 10 μm. (c) Normalized biomass of *E. coli* cells on upright flat PDMS and in the wells of S10 D5 and S10 D2 patterns. ***, *p* < 0.001.

Adhesion (4 h) of the red fluorescent *E. coli* RP437/pRSH103 on the library patterns was tested under static conditions (Figure 2a) and with agitation (shaking at 200 rpm: Figure S3). Most features had similar biomass to the flat control (red bar). However, three outliers from the PDMS library showed up to 2 times greater biomass than the flat control (*p* < 0.05, one-way ANOVA adjusted by Tukey’s test). The three outliers from the PDMS library were S5 D2, S10 D2, and S10 D5 [S, feature side length (μm); D, distance between features (μm)]. Under agitation, no significant difference in biomass was observed among the PDMS library patterns (*p* > 0.05, one-way ANOVA adjusted by Tukey’s test). When the surface was turned upside down and the number of adherent cells was measured over time (data not shown), the results were similar to the upright patterns under the static condition, confirming that the results were indeed adhesion rather than random cell settling due to gravity. To corroborate the high-throughput screening results, images of two outliers of the library patterns (S10 D2, S10 D5) were obtained using confocal microscopy (Figure 2b). The normalized biomass inside the features was calculated using COMSTAT^23^ and compared with the flat control (Figure 2c). The biomass on flat (0.07 ± 0.01 μm^3^/μm^2^), S10 D5 (0.73 ± 0.05 μm^3^/μm^2^), and S10 D2 (0.50 ± 0.02 μm^3^/μm^2^) showed a trend similar to that obtained from the high-throughput method.

**Figure 3:**
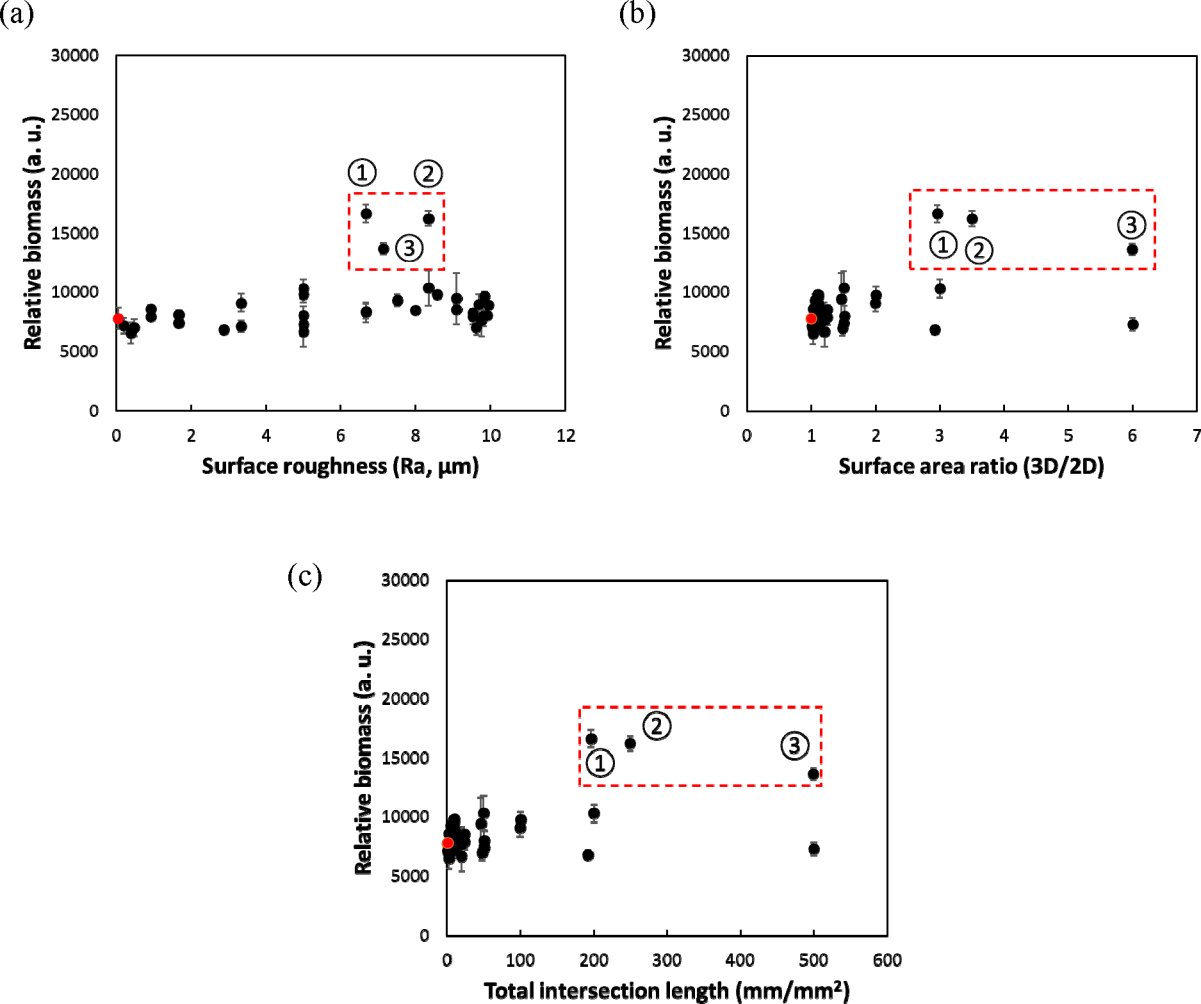
Relative biomass of *E. coli* RP437/pRSH103 vs (a) surface roughness, (b) surface area ratio, and (c) total intersection length, after 4 h attachment on the PDMS surfaces under static condition. Three outliers are highlighted in the dotted red rectangles: red dots, flat control; ① S10 D5; ② S10 D2; ③ S5 D2.

To further understand the correlation between surface topography and bacterial adhesion, the biomass was plotted vs surface roughness (Figure 3a), surface area ratio (Figure 3b), and intersection length (sum of bottom side lengths of recessive square patterns; Figure 3c). There was little difference in biomass across a wide range of surface roughness levels (0–10 μm, *R*_a_) (Figure 3a), and there were three outliers that had significantly greater biomass than others at the same level of roughness (S5 D2, S10 D2, and S10 D5). Thus, surface roughness was not an effective parameter for predicting bacterial adhesion. In comparison, plotting biomass vs surface area ratio or pattern intersection length showed clusters of patterns on the left of plots (low biomass on surfaces with low 3D/2D ratio and small pattern size). These parameters also did not provide a clear trend line for prediction of outliers (Figure 3). Overall, these findings emphasize the importance of specific patterns and caution against making prediction with an overly generic parameter.

Previous studies^25,26,36−39^ have shown that surface pattern size in the range of 5–20 μm affect bacterial adhesion. Given the size of *E. coli* cells used in this study (2–3 μm long) and the surface appendages such as flagella, it would be challenging to enter 2 μm cubic features. Consistently, the cells were found to primarily adhere to the flat top surface, resulting in reduced overall adhesion when compared with flat surfaces.

### 3.4 Real-Time Study of Adhesion Using Confocal Microscopy

During our tests, bacteria exhibited preferential adhesion to the edges of the recessive cubes (S10 D5 and S10 D2; Figure 2 and Video S1). As shown in the Video S1, initial attachment of *E. coli* RP437/pRSH103 at the edges of the recessive cubes was observed and an increased number of adjacent cell attachments due to either cell proliferation or adhesion of additional cells was seen over time.

**Figure 4:**
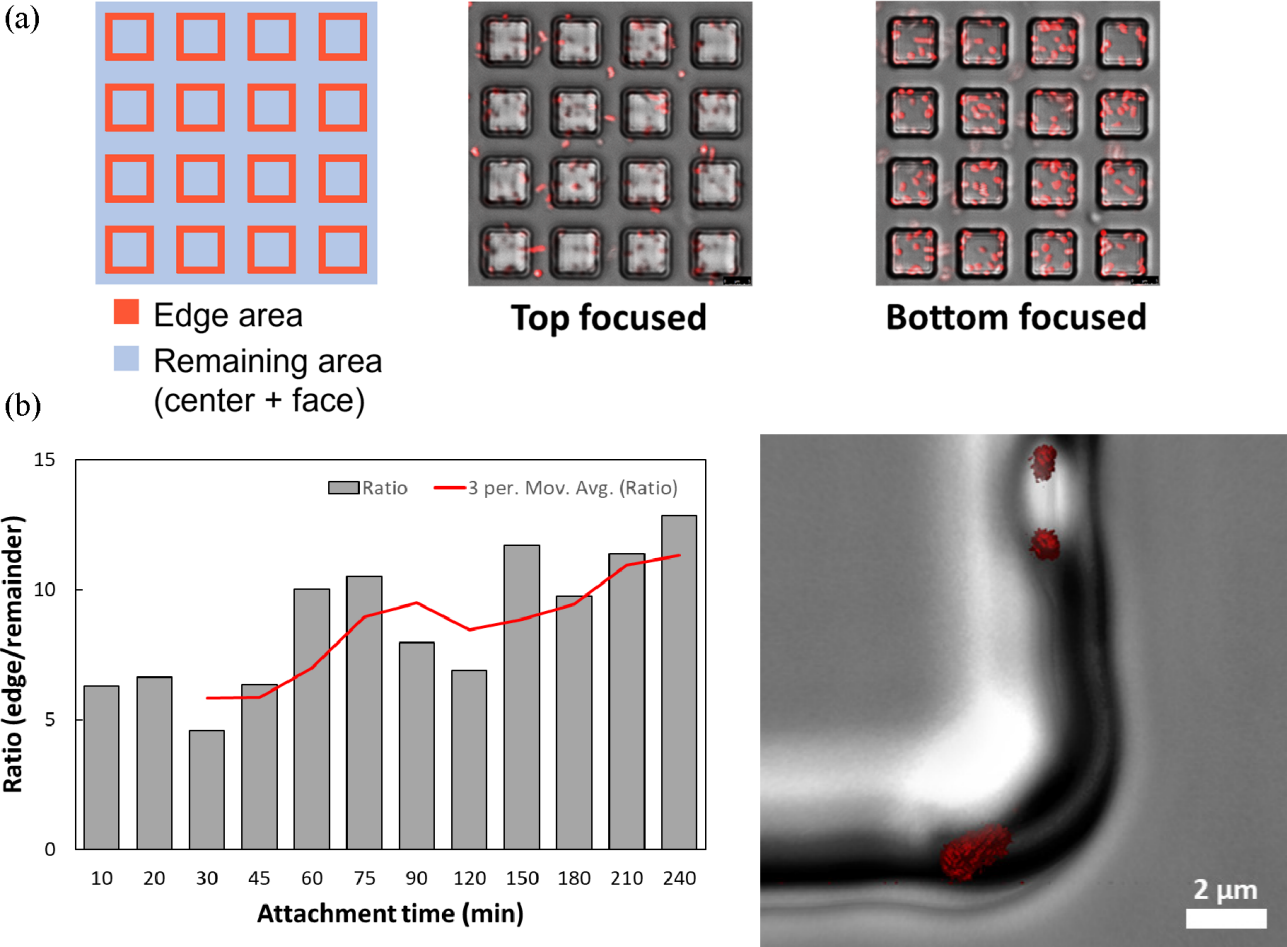
Confocal analysis of *E. coli* RP437/pRSH103 cell attachement. (a) Schematic illustration of edge area (36 μm^2^/feature, red) and the remainder (189 μm^2^/feature, blue), as well as top- and bottom-focused confocal fluoresence microscopic images. (b) Ratio of cells attached to the edge vs remainder areas (normalized by surface area), which increased over the first 4 h of incubation. (c) 3D confocal Z-stack reconstruction of fluorescent *E. coli* RP437/pRSH103 cells attached in the edge area. Scale bar = 2 μm.

Real-time confocal microscopy was used to further study time-dependent bacterial adhesion in these recessive patterns focusing on the outlier S10 D5 since it has more biomass than most of the other features (Figure 4). The number of the attached cells, normalized by the surface area, was used to calculate an attachment at the “edge” vs the “remainder” of a feature (Figure 4a). The edge is referred to as the area within 1 μm from all four directions of the inside feature walls, while the remainder includes inner center area and interfeature area excluding the edge defined above. Confocal images taken during 4 h attachment (Figure S4) showed that the ratio of cells attached at edge/remainder area increased by 2-fold over time (Figure 4b). Interestingly, some first-layer cells oriented vertically (in the *z*-plane) rather than horizontally along the walls of recessive patterns (Figure 4c). New cells emerged after division were observed growing from attached parent cells (Video S1). The confocal microscopy procedures were done here manually to gain insight into the attachment process and are not required for quantitative analysis of biomass to be performed in a high-throughput manner as demonstrated above.

### 3.5 Biofilm Growth over 24 h

**Figure 5:**
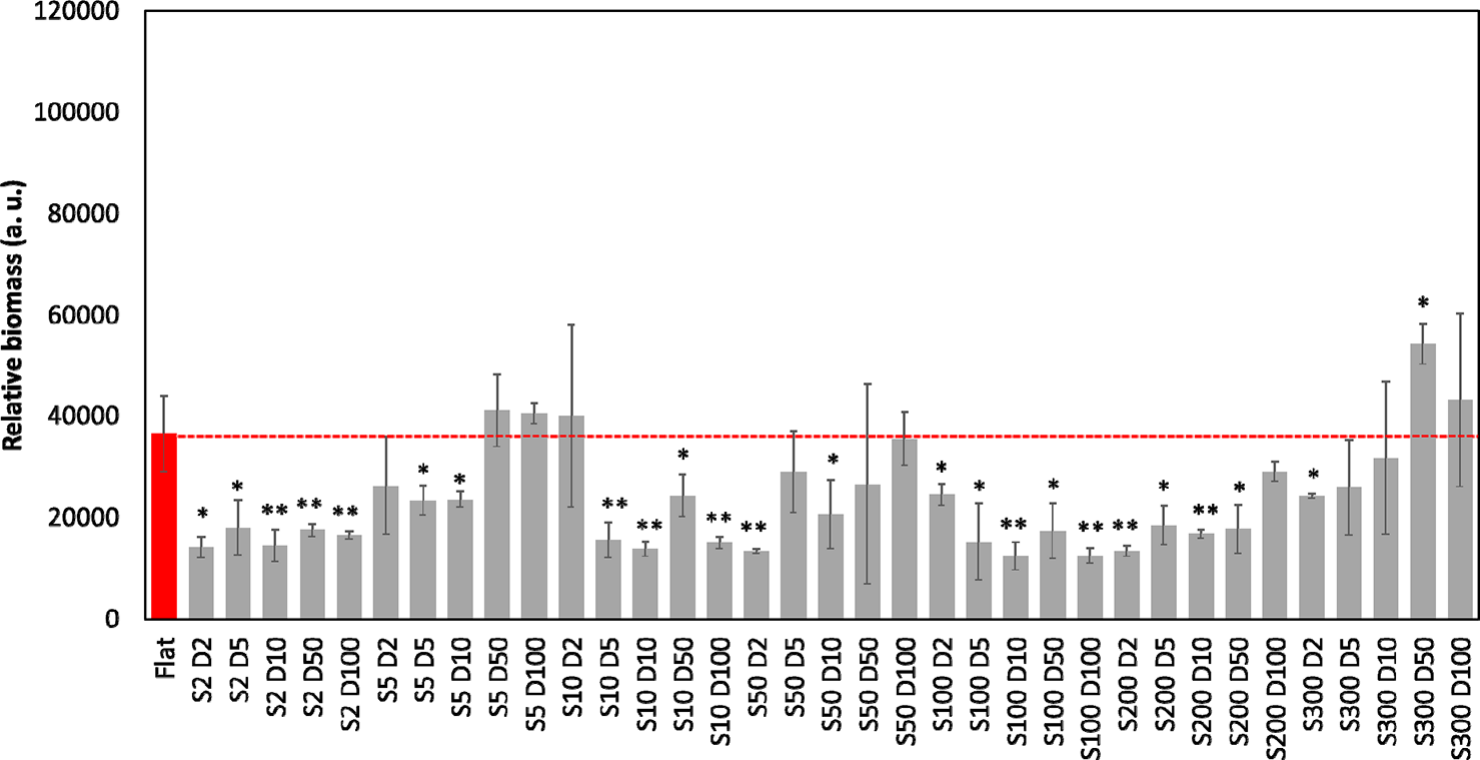
Relative biomass of *E. coli* RP437/pRSH103 after 24 h biofilm growth on patterned PDMS surfaces under static condition. Red, flat control. *, *p* < 0.05; **, *p* < 0.01.

Next the high-throughput assay was used to study 24 h biofilm growth on the library patterns. About 5 times more fluorescence signal from biomass was observed on the smooth surface at 24 h (Figure 5) than at 4 h. In contrast to the 4 h adhesion results, the three outliers (S5 D2, S10 D2, and S10 D5) from the pattern library at 24 h did not exhibit significantly higher biomass compared to the rest of the library patterns. This indicates that static surface topography may play a bigger role in initial bacterial adhesion than long-term biofilm growth. Materials that can prevent the attachment of pathogens but promote host cell integration may reduce the risk of infection.

## 4 Discussion

Most current in vitro methods for testing bacterial adhesion on medical device materials are low throughput, with the exception of some microplate-based methods (e.g., those based on crystal violet staining) and the Calgary biofilm device.^40^ However, current microplate-based methods require manual wash, and reproducibility is a challenge due to the lack of control over rinsing forces and the inherent variability in scraping, plating, and culturing cells. The Calgary device is designed for high-throughput screening of antibiotic susceptibility of biofilms, but not biomaterials with different topographic features. This work first addressed these limitations through development of a high-throughput microplate test for adhesion and biofilm formation on different material samples, including controllable and reproducible rinsing by a plate washer. The use of a plate washer reduced the labor and time involved with manual rinsing, while also providing advantages of reproducibility and control (shear force at a level that thoroughly removed loosely bound cells but not those which were firmly attached).

Computational simulation verified the range of shear forces during rinsing and ruled out the possibility of excessive shearing or insufficient flushing that might be produced by the rinsing process. The use of a plate reader to quantify fluorescence of RFP producing *E. coli* allowed for rapid and sensitive measurement of the relative bioburden on the materials. This assay was then used to study bacterial interaction with micrometer-sized topographic patterns relevant to textured breast implants. While there has been an association between textured breast implants and incidence of BIA-ALCL, the etiology is still not understood. Analysis of explants is challenging, and results have been inconsistent. Animal models, while hinting at the possible importance of materials compatibility,^41^ have not yet provided a causative mechanism. Recently, the contribution of bacterial biofilms in disease pathogenesis has been one of the major hypotheses discussed by clinical researchers. In the present study, the high-throughput method was applied to better understand how common breast implant texture features may affect adhesion or biofilm formation.

Due to throughput limitations, many prior in vitro studies of topography have been informative but have used relatively small libraries of surfaces, resulting in limited validation of trends across multiple size ranges. The high-throughput capability of our method allowed us to test a large library designed with the goal of separating the influence of qualitative features from surface roughness. To do this, the side length and distance were purposefully varied to obtain different patterns with the same surface roughness. For example, S2 D2, S5 D5, S10 D10, S50 D50, and S100 D100 have the same surface roughness (*R*_a_), 5 μm. Similarly, S10 D5, S100 D50, and S200 D100 have the same surface roughness of 6.67 μm and S5 D10 and S50 D100 have the same surface roughness of 3.33 μm. The square-like recessive features tested in this study do not include overhangs or pores which are observed in optical microscopic images and scanning electron microscopic images of some commercial implants.^3,24,41−43^

The library size and analytical characteristics of this approach enabled us to negate the hypothesis that roughness, a single parameter of the material, can be used to predict bacterial adhesion. Pattern size and 3D/2D ratio revealed less biomass on certain surfaces with small patterns but were not able to provide good prediction across the range of patterns tested especially when features became larger. In addition, real-time confocal imaging of the surfaces showed that the number of cells adhering to the edge area vs the center increased over time. Taken together, these results point to the importance of both size and qualitative feature type (i.e., internal edges) in early adhesion behavior, which may be related to the size of the *E. coli* flagella and their roles in early biofilm formation.^44^ In summary, screening of a relatively large pattern library allowed us to better understand bacterial attachment to different topographies than low-throughput methods with just a few representative patterns. Future tests using this format can be designed to mimic the in vivo environment of different biomaterials more closely, which may add additional insight into how physiological parameters affect early stage bacterial adhesion.

The results of this work also indicate that the use of surface area alone is inadequate to estimate propensity for bacterial adhesion and biofilm in vitro. Predication based solely on this factor can be misleading, and other factors of specific topography need to be considered. For commercial patterns that have overhangs and pores and those with nano- or micrometer-scale irregularities, these features may drive phenotypic changes toward permanent adhesion and colonization. The method developed here can be used to further study the contribution of these additional parameters to early stage bacterial adhesion. This method can also be adapted for use with other strains and materials through adjustment of rinsing conditions.

## 5 Conclusions

In summary, a high-throughput assay was developed to quantitatively compare biofilm formation on biomaterial surfaces. The assay has advantages over conventional low-throughput assays or those that require manual rinsing. The uniquely high throughput and reproducibility of the assay allowed for study of topographic features such as those relevant to textured breast implants. The results caution against making assumptions that are too broad based solely on a quantitative measurement such as surface area or roughness. Correlations between bacterial adhesion/biofilm and roughness may only be valid for a very small subset of size scales and topographic patterns. These findings highlight the importance of additional work to study how bacteria interact with specific qualitative types of surface features and patterns at multiple size scales, which may be found on medical devices. It is also essential to determine how their interaction varies over time during the critical period between 4 and 24 h, including how topography affects biological signaling and phenotypic changes that lead to biofilm formation. The high-throughput approach developed in this work can help to facilitate further studies needed to answer these questions.

## Supporting Information Available

Additional experiment and method details (Figures S1 and S2); relative biomass results (Figure S3); representative confocal microscopic images of patterns (Figure S4); key parameters (surface roughness, surface area ratio, and total intersection length) (Table S1) (PDF)
Video S1 4 h tracking of *E. coli* RP437/pRSH103 attachment on the S10 D5 “facing down” surface in LB medium (AVI)

## Supplementary Material

mt2c00367_si_001.pdf

mt2c00367_si_002.avi
