# Supplementary material for: High-Throughput Biofilm Assay to Investigate Bacterial Interactions with Surface Topographies
Source: ACS Appl Bio Mater. 2022 Jul 11;5(8):3816–25. doi: 10.1021/acsabm.2c00367 (PMC9382637; doi:10.1021/acsabm.2c00367)
Supplement: Supplementary file 1 — mt2c00367_si_001.pdf [file mt2c00367_si_001.pdf]

Supporting Information

A high-throughput biofilm assay to investigate bacterial interactions with surface topographies

Sang Won Lee[1,2], Erick L. Johnson[3], J. Alex Chediak[2,4], Hainsworth Shin[2], Yi Wang[2], K. Scott Phillips[2], and Dacheng Ren[1,5,6*]*

[1]Department of Biomedical and Chemical Engineering, Syracuse University, Syracuse, NY 13244, United States

[2]United States Food and Drug Administration, Office of Medical Products and Tobacco, Center for Devices and Radiological Health, Office of Science and Engineering Laboratories, Division of Biology, Chemistry, and Materials Science, Silver Spring, MD 20993, United States

[3]Mechanical and Industrial Engineering, Montana State University, Bozeman, MT 59717, United States

[4]Department of Mathematical Sciences, California Baptist University, Riverside, CA 92504, United States

[5]Department of Civil and Environmental Engineering, Syracuse University, Syracuse, NY 13244, United States

[6]Department of Biology, Syracuse University, Syracuse, NY 13244, United States

*Corresponding authors:

Dacheng Ren: Phone +1-315-443-1257. Fax +1-315-443-9175. Email : dren@syr.edu

K. Scott Phillips: Phone +1-301-796-0238. Fax +1-301-796-9826.

Email : Kenneth.Phillips@fda.hhs.gov

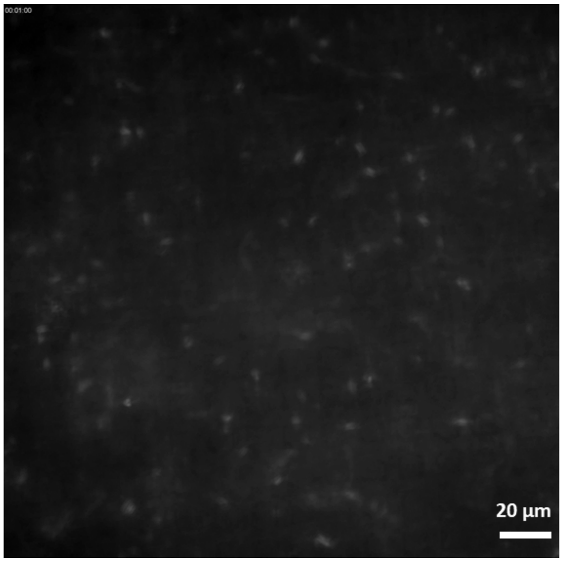

Video S1. A video tracking of *E. coli* RP437/pRSH103 4 h attachment on S10 D5 ‘facing down’ surface in LB medium. Scale bar = 20 µm.

(a) (b)

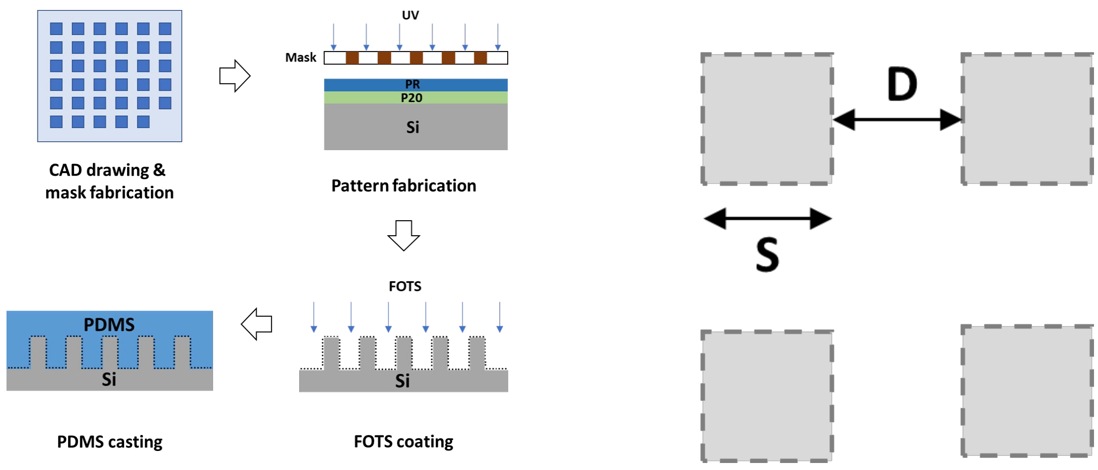

Figure S1. (a) Schematic of patterned PDMS fabrication. A combination of features was drawn by CAD software, L-edit; and a quartz mask was fabricated based on the design. P20 (an adhesion layer) and a photoresist (PR) layer were deposited by a spin coater, exposed 1:1 contact photolithography, and etched by an etcher to create features. A fluorooctyltrichlorosilane (FOTS) layer was then deposited to make the surface hydrophobic. Lastly, polydimethylsiloxane (PDMS) was cast using the patterned Si master as a mold. (b) A schematic of patterns with specific side length (S) and distance (D) between features. S varied between 2 μm to 300 μm and D varited between 2 μm and 100 μm.

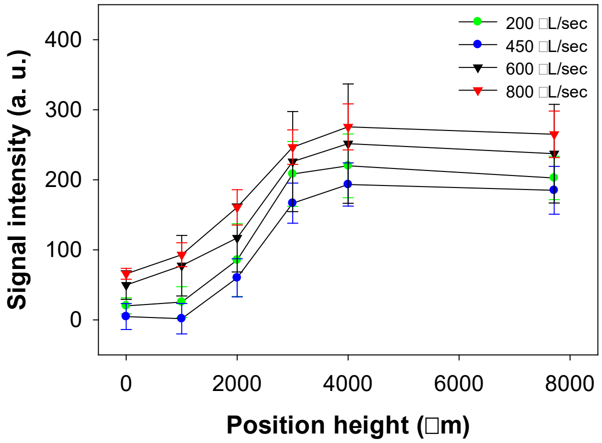

Figure S2. Red fluorescent signal intensity was measured by a plate reader with varying focal position height from 0 μm (bottom) to 8000 μm (top) of the well. The dispense flow rate of PBS solution varied from 200 μL/s to 800 μL/s.

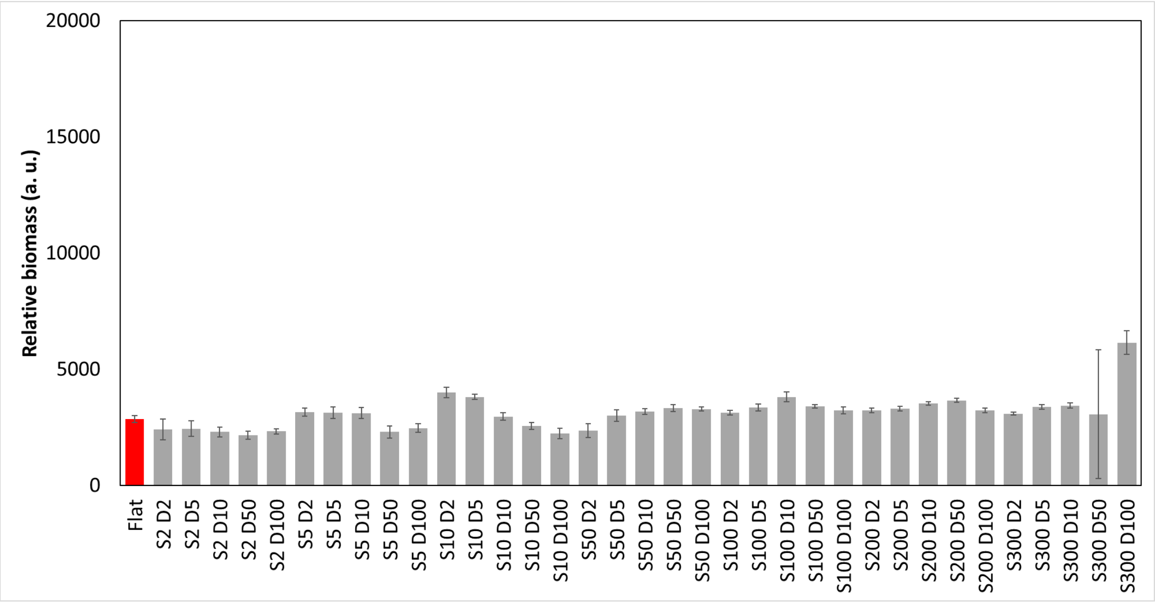

Figure S3. Relative biomass of *E. coli* RP437/pRSH103 after 4 h attachment on the PDMS surfaces with agitation (shaking at 200 rpm). Red bar: flat control.

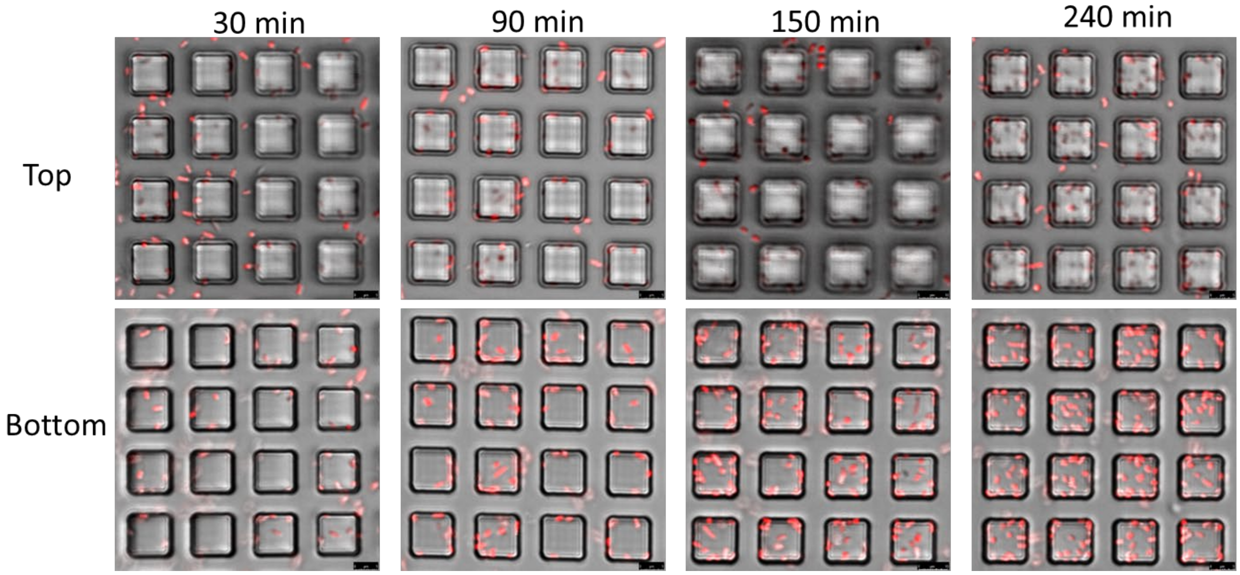

Figure S4. Representative confocal microscopic images of patterns (top and bottom focal point) with the attached cells after varying amount of attachment time: 30, 90, 150, and 240 min. Scale bar = 5 µm.

Table S1. Key parameters of PDMS library samples including surface roughness, surface area ratio, and total intersection length. S: side length of the features. D: distance between features.

PDMS samples	Distance (D; µm)														
	2			5			10			50			100		
	Surface roughness (µm)	Surface area ratio	Total intersection length (mm/mm²)	Surface roughness (µm)	Surface area ratio	Total intersection length (mm/mm²)	Surface roughness (µm)	Surface area ratio	Total intersection length (mm/mm²)	Surface roughness (µm)	Surface area ratio	Total intersection length (mm/mm²)	Surface roughness (µm)	Surface area ratio	Total intersection length (mm/mm²)
Side (S; µm) 2	5	6	500.00	2.86	2.92	19.22	1.67	1.50	50.31	0.38	1.03	2.51	0.20	1.01	0.95
5	7.14	5.99	499.28	5	3	200.00	3.33	1.99	99.41	0.91	1.05	5.07	0.48	1.02	2.00
10	8.33	3.50	249.90	6.67	2.96	196.00	5	2	100.00	1.67	1.10	10.24	0.91	1.03	2.56
50	9.62	1.48	48.05	9.09	1.46	46.21	8.33	1.50	49.93	5	1.2	20.00	3.33	1.10	10.08
100	9.80	1.51	51.08	9.52	1.24	24.34	9.09	1.24	24.34	6.67	1.20	19.60	5	1.1	10.00
200	9.90	1.2	20.00	9.76	1.2	20.00	9.52	1.2	20.00	8	1.11	10.95	6.67	1.09	9.25
300	9.93	1.11	10.80	9.84	1.10	10.09	9.68	1.11	10.80	8.57	1.10	10.09	7.5	1.05	5.29
